# A Study on Non-Linear DPL Model for Describing Heat Transfer in Skin Tissue during Hyperthermia Treatment

**DOI:** 10.3390/e22040481

**Published:** 2020-04-22

**Authors:** Sunil Kumar Sharma, Dinesh Kumar

**Affiliations:** 1 College of Computer and Information Sciences, Majmaah University, Majmaah 11952, Saudi Arabia; 2 Govt. Polytechnic Nawada, Bihar 805122, India; dineshaukumar@gmail.com

**Keywords:** blood perfusion, DPLBHT model, FERK (4,5) method, Gaussian external heat source, hyperthermia treatment, metabolic heat source, non-linear, skin tissues

## Abstract

The article studies the simulation-based mathematical modeling of bioheat transfer under the Dirichlet boundary condition. We used complex non-linear dual-phase-lag bioheat transfer (DPLBHT) for analyzing the temperature distribution in skin tissues during hyperthermia treatment of infected cells. The perfusion term, metabolic heat source, and external heat source were the three parts of the volumetric heat source that were used in the model. The non-linear DPLBHT model predicted a more accurate temperature within skin tissues. The finite element Runge–Kutta (4,5) (FERK (4,5)) method, which was based on two techniques, finite difference and Runge–Kutta (4,5), was applied for calculating the result in the case of our typical non-linear problem. The paper studies and presents the non-dimensional unit. Thermal damage of normal tissue was observed near zero during hyperthermia treatment. The effects of the non-dimensional time, non-dimensional space coordinate, location parameter, regional parameter, relaxation and thermalization time, metabolic heat source, associated metabolic heat source parameter, perfusion rate, associated perfusion heat source parameter, and external heat source coefficient on the dimensionless temperature profile were studied in detail during the hyperthermia treatment process.

## 1. Introduction

Tumors or cancerous cells are a classical sign of inflammation and can be benign or malignant (cancerous). In America, nearly 606,880 people were anticipated to die from cancer in 2019, which translated to about 1660 deaths each day. Cancer is the second most common cause of death in the U.S., exceeded only by heart disease [1]. Therefore, the study of tumor treatment is required to save human lives in the world. Several researchers [2,3,4,5] studied the therapeutic treatment of bioheat transfer in skin tissue with the help of mathematical modeling. Mathematical modeling of heat transfer in biological systems has been a broad field of study for various biologists, physicians, mathematicians, and engineers [6]. An efficient clarification of the physiological relation between the vascular system and tissue is necessary in medical science for treating fatal diseases like tumors. Currently, mathematical models are commonly used to describe the process of hyperthermia, cryosurgery, and many other techniques for the treatment of tumors. It is mandatory to know the thermal effect in skin tissue during the hyperthermia treatment process. The size, shape, and location of tumors are important factors for the treatment process [7].

Several bioheat transfer-based models have assumed the physiological properties of human beings to be constant, which are not described accurately for hyperthermia treatment of tumors or cancer. However, because the inner structure of the human body is inhomogeneous, the physiological parameters depend on local tissue temperature. Some researchers [8,9,10] considered the perfusion term to be a function of the temperature in local tissue. Similarly, some authors [11,12] assumed a metabolic heat source in their model, and this was also a function of the temperature in local tissue. The perfusion term and metabolic heat source are both considered a function of temperature in local tissue, i.e., a realistic-type function, and the external heat source is taken as electromagnetic radiation [13]. However, the location and shape parameters are not derived very well.

Modeling of the tumor treatment is done by the study of the heat transfer in the biological system. The treatment of tumors has been broadly studied in pre-clinical models with human clinical trials [14]. The treatment techniques such as hyperthermia, thermal ablation, cryoablation, and cryosurgery are used for selectively destroying the tumor in living skin tissue. Thermal therapy is an ideal modality for the treatment of infected cells using different types of external heat sources like electromagnetic irradiation [6,10,12,13], magnetic nanoparticles (MNPs) [15,16], etc.

Andreozzi et al. (2019) [17] performed a sensitivity analysis of the hyperthermia effects on a typical transient percolation process in a tumor. In this process, the temperature was raised in a tumor region according to different categories of thermal therapy. Bioheat transfer was analyzed by Andreozzi (2019) [18], who took many mechanisms into account, such as thermal conduction in tissues, convection and blood perfusion, metabolic heat generation, vascular structure, and the change of tissue properties depending on the physiological condition. A numerically investigated bioheat transfer model has been used for hyperthermia treatment with the convection term instead of the perfusion term in the energy conservation equation for tissue and blood [19]. Wang et al. (2015) [20] studied the temperature distribution within biological organs for therapeutic aspects related to hyperthermia treatments such as radiofrequency ablation. The accuracy of temperature-based treatment depends on accurate prediction and control of the temperature in skin tissue [12]. A quantitative analysis of the relationship between arterial blood and tissue temperature was done by Pennes (1948) [21]. There are many bioheat transfer models for studying the heat transfer in skin tissue in the existing literature, and it was found that the commonly used bioheat transfer model for analyzing the temperature distribution is the Pennes bioheat transfer model [21], which is based on the classical constitutive relation that was introduced by Fourier, i.e.,

(1) q(r,t)=▿T(r,t).

Penne’s bioheat transfer (PBHT) model predicts the temperature with the infinite speed of propagation, which is incompatible in the real domain. To unify this, consecutively, Cattaneo [22] and Vernotte [23] introduced it in 1958, independently; so, the heat flux and temperature gradient are combined with a constitutive relation, which is given as:

(2) $$q\left(r,t+\tau_{q}\right)=▿T\left(r,t\right).$$

Equation (2) is known as the single-phase-lag (SPL) constitutive relation. Relaxation time $\left(\tau_{q}\right)$ indicates the lag time due to heat flux. In 1995, Tzou [24,25] introduced his concept in the generalization of the SPL model by assuming thermalization time due to the temperature gradient, called the dual-phase-lag (DPL) constitutive relation, i.e.,

(3) $$q\left(r,t+\tau_{q}\right)=▿T\left(r,t+\tau_{T}\right).$$

where $\tau_{T}$ is known as the thermalization time, and the combination of the DPL constitutive relation and energy balance equation is known as the DPLBHT model. For the study of micro-scale responses in time and to capture the micro-scale responses in space, the DPL bioheat transfer model has been used. Therefore, in the existing literature, the DPL bioheat transfer model is the most realistic in comparison with others [26].

Thermal-probes and cryoprobes are used for tumor or cancer treatment. In the DPLBHT model, the thermal correlation between the cylindrical cryoprobe and skin tissues was studied by Mochnaki and Machrzak [27]. A relation was developed between the heat transfer in perfused skin tissue with the thermal-probe and a local symmetric component of the vascular system [28]. Many authors [8,29,30,31,32,33] have assumed a non-linear PBHT model with a physiological property such as perfusion rate for finding the temperature in skin tissues. In reality, These types of models do not give realistic data of the temperature in skin tissue because they do not consider the relaxation and thermalization time in the heat flux and temperature gradient, respectively. This is a drawback of this type of nonlinear bioheat transfer model.

The thermal behaviors of a perfused tissue with two co-current and counter-current vascular networks were investigated numerically under an interstitial hyperthermia process using both local thermal equilibrium (LTE) and local thermal nonequilibrium (LTNE) assumptions [34]. Zhang et al. [30] analyzed the PBHT model under the steady-state condition with the perfusion rate in skin tissues varying linearly, quadratically, and exponentially with local skin tissue temperature and solved it using the boundary reciprocity method. The smoothed particle hydrodynamic method was used for the results of the nonlinear PBHT model with space coordinate-dependent thermal conductivity. Several researchers studied the temperature distribution in skin tissues using the DPL bioheat transfer model, undertaking different types of volumetric heat sources. They solved the DPLBHT model using the finite element Legendre wavelet Galerkin method [15] and used the FERK (4,5) method [10,12,13] and finite difference-decomposition method [6]. The development of the reconfigurable distributed multiple-input multiple-output technique in a practical communication environment was proposed by Do and Haas [35].

In this paper, we propose the highly non-linear DPLBHT equation under the constant boundary condition, which consists of temperature-dependent metabolism and blood perfusion heat generation, as well as a Gaussian heat source. This model is very useful for hyperthermia treatment because the physiological properties of biological skin tissue are considered as a realistic function of local tissue temperature. These types of physiological properties with a Gaussian heat source have been used in the DPLBHT model till now. Due to the lower computational complexity and less data storage, combined with the high accuracy, the problem is converted into a system of ordinary differential equations with initial conditions using the finite difference scheme. This system of ordinary differential equations with initial conditions is solved using the RK (4,5) scheme. All parameters such as the location parameter, regional parameter, and relaxation and thermalization time provide a better understanding of the control temperature in the hyperthermia condition. The metabolic heat source, associated metabolic heat source, and external heat source increase as the local skin tissue temperature increases. The perfusion rate, associated with the perfusion heat source parameters, decreases as the local skin tissue temperature increases as well.

This paper is organized into seven sections. In the first section, the introduction of the bioheat transfer models, some methods, and also the nomenclature are given, which support our proposed work. Our mathematical problem is formulated in Section 2. In the third section, our mathematically formulated problem is converted into a dimensionless form. Section 4 describes the solution of the proposed problem using the FERK (4,5) method. In Section 5, we propose the exact solution of our problem for a particular case to verify the FERK (4,5) method. The results and discussion are given in Section 6. The last section consists of the conclusions of the proposed work.

## 2. Formulation of the Problem

**Figure 1:**
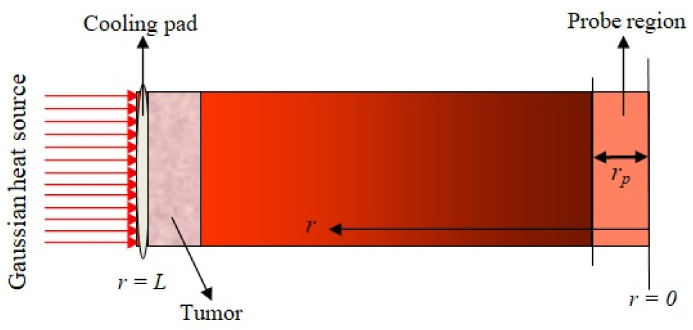
Schematic geometry of the skin tissue with the tumor and probe region.

Hyperthermia is a treatment process of tumors and cancer. In this treatment process, the temperature of the tumor region is kept between 41 °C and 46 °C, with approximately a time period of 15 to 60 min [36]. The outer surface of the skin tissue is kept at a fixed temperature $T_{0}$ = 37 °C, initially, during hyperthermia treatment. The outer surface is heated with a Gaussian heat source externally. The inner surface of the skin, i.e., r=0, is insulated, and the temperature of the outer surface is maintained constant with the help of a cooling pad, which is shown in Figure 1. The tumor region is indicated by the schematic geometry of the skin tissue, which depends on the probe region parameter $r_{p}$. If this parameter increases or decreases, then the location of the tumor in skin tissue changes. This is seen in the external heat source. We used a heated metal disc with temperature control and approximated by the one-dimensional non-linear DPLBHT model in the Cartesian coordinate system under the first kind (constant) boundary condition. This is a combination of the DPL constitutive relation and also the one-dimensional energy balance equation. The energy balance equation in one-dimensional form is written as [21]:

(4) $$\rho c\frac{\partial T(r,t)}{\partial t}=-\nabla q\left(r,t\right)+Q_{b}+Q_{m}+Q_{r},$$

where the left-hand side denotes the conduction term in the skin tissue and the first term of the right-hand side denotes the convection term in the skin tissue. $Q_{b}$, $Q_{m}$, and $Q_{r}$ are the perfusion heat source, the metabolic heat source, and the externally applied heat source term, which is taken as a Gaussian-type heat source. The metabolic heat source is generated in the body by the intake of food, and the perfusion heat rate is the heat source that is spent in blood circulation. Still, the Gaussian heat source is externally applied on the outer surface.

The blood perfusion $Q_{b}$ indicates convection in the blood. This term removes heat due to the flow of blood. It was defined by several researchers [10,13,30].

(5) $$\;Q_{b}=\;w_{b}\left(T\right)\rho_{b}c_{b}\left(\;T_{b}-T\right),$$

The perfusion coefficient $w_{b}\left(T\right)$ depends on the tissue temperature due to the anatomical structure of the skin tissue containing blood vessels. $w_{b}\left(T\right)$ was taken from Zhang et al. [30]:

(6) $$\;w_{b}\left(T\right)=\;w_{b0}\times e^{a\left(\frac{T-T_{0}}{T_{0}}\right)},$$

where $w_{b0}$ and *a* are the assumed blood perfusion coefficient and associated blood perfusion coefficient initially.

In Equation (4), $Q_{m}$ indicates the metabolism of the human body. It is the factor that increases the local tissue temperature. The realistic function $Q_{m}$ of the local tissue temperature was taken from Mitchell et al. [11]:

(7) $$Q_{m}=Q_{m0}\times 2^{\beta \left(\frac{T-T_{0}}{10}\right)},$$

where $Q_{m0}$ and β are the initial metabolic heat source coefficient and the associated metabolic constant, respectively. *T* and $T_{0}$ are the local skin temperature and the initial temperature of the skin tissue, respectively.

A Gaussian heat source-type expression is curve fitted based on the experimental measurements of the specific absorption rate distribution in the target region [37]. The selection of a Gaussian distribution source termed as a spatial heating source helps to determine the hyperthermia position. Therefore, that Gaussian-type heat source expression is applied on the outer surface, and it was taken from Kumar et al. [13]:

(8) $$Q_{r}=Q_{r0}\;e^{\eta {\left(\bar{r}-r_{p}\right)}^{2}},$$

where $Q_{r0}$ is the reference value of external heat generation in the tissue; $\bar{r}\;\left(=L-r\right)$ is the measured length from the outer surface; *L* is the length of the tissue temperature. $r_{p}$ is the length of the probe region.

Now, the energy balanced equation is combined with the approximation of the DPL constitutive relation of the first order in one-dimensional form, then we obtain the non-linear DPLBHT equation [13], i.e.,

(9) $$\begin{array}{c} \rho c\tau_{q}\frac{\partial^{2}T}{\partial t^{2}}+\rho c\frac{\partial T}{\partial t}=k\frac{\partial^{2}T}{\partial r^{2}}+k\tau_{T}\frac{\partial^{3}T}{\partial t\partial r^{2}}+\;Q_{b}\\ +\;Q_{m}+\;Q_{r}+\tau_{q}\frac{\partial Q_{b}}{\partial t}+\tau_{q}\frac{\partial Q_{m}}{\partial t}+\tau_{q}\frac{\partial Q_{r}}{\partial t}, \end{array}$$

initially subjected to:

(10) $$T\left(r,0\right)=\;T_{0},\;\;\frac{\partial T(r,0)}{\partial t}=0.$$

Physically, the heating/cooling condition at the outer surface r=L was given in [15], i.e.,

(11) $$T\left(L,t\right)=\;T_{w},$$

and the inner surface is adiabatic [10,12], so that it is defined as:

(12) $$\frac{\partial T(0,t)}{\partial r}=0.$$

In the existing literature, the DPLBHT model with a Gaussian heat source in the presence of a realistic function of the metabolic and perfusion heat generation terms has not been studied till now. We considered the Gaussian heat source because it helps with the control of temperature during hyperthermia treatment.

## 3. Conversion of the Problem into Dimensionless Form

One way of breaking away from the quantitative features of the corresponding model is to rewrite the equation in terms of non-dimensional quantities. The proposed DPLBHT equations reduces in terms of non-dimensional quantities. These types of studies allow an easy and direct comparison of the proposed mathematical model. Therefore, the non-dimensionless variables with similarity criteria was introduced in [13]:

(13) $$\begin{array}{c} x=\frac{r}{L},F_{o}=\frac{kt}{\rho cL^{2}},F_{oq}=\frac{k\tau_{q}}{\rho cL^{2}},F_{oT}=\frac{k\tau_{T}}{\rho cL^{2}},\\ \theta =\frac{T-T_{0}}{T_{0}},P_{f}=\sqrt{\frac{w_{b0}c_{b}\rho_{b}}{k}}L,P_{m}=\frac{Q_{m0}L^{2}}{kT_{0}},\\ P_{r}=\frac{Q_{r}}{kT_{0}}L^{2},\alpha =0.1\times T_{0}\times \beta ,\eta_{1}=\eta \times L,\;\\ \bar{x_{p}}=1-x_{p}.\; \end{array}$$

Upon using Equation (13), then Equations (9)–(12) can be reduced as follows:

(14) $$\begin{array}{c} F_{oq}\frac{\partial^{2}\theta }{\partial F_{o}^{2}}+\Theta \frac{\partial \theta }{\partial F_{o}}=\frac{\partial^{2}\theta }{\partial x^{2}}+\;F_{oT}\frac{\partial^{3}\theta }{\partial F_{o}\partial x^{2}}+P_{f}^{2}\left(\theta_{b}-\theta \right)\;e^{a\theta }\\ +\;P_{m}\times 2^{\alpha \theta }+P_{r}\exp\left(\eta_{1}{\left(\bar{x}-x_{p}\right)}^{2}\right),\; \end{array}$$

where $\Theta =\left[1-\left(a\left(\theta_{b}-\theta \right)-1\right)F_{oq}P_{f}^{2}\;e^{a\theta }-F_{oq}P_{m}\alpha \log\left(2\right)\cdot 2^{\alpha \theta }\right]$, initially subjected to:

(15) $$\theta \left(x,0\right)=0\;\;\mathrm{and}\;\;\frac{\partial \theta (x,0)}{\partial F_{o}}=0.$$

The outer boundary condition:

(16) $$\theta \left(1,Fo\right)=\theta_{w},$$

and the inner boundary condition is taken as insulated:

(17) $$\frac{\partial \theta (0,F_{o})}{\partial x}=0.$$

Our problem transformed into dimensionless form is solved using the finite element Runge–Kutta(4,5) method. The detail of this method is described in the following section.

## 4. Finite Element Runge–Kutta (4,5) Method

The FERK (4,5) method consists of two techniques: (i) discretization in space coordinates using the finite difference scheme [38]; then our proposed problem is reduced to a system of ordinary differential equations (ODEs); and (ii) another scheme, RK (4,5) [39], is used for the solution of the ODEs. These are described in the following subsection.

### 4.1. Discretization Technique in Space Coordinates

We discretize the finite range of space coordinates [0,1] into l+1 discrete points, i.e., $0=x_{0}<x_{1}<x_{2}<x_{3}<\cdots <x_{i}<\cdots <x_{l}<x_{l+1}=1$, where $x_{i+1}=x_{i}+h$, and *h* is the constant length of the subinterval of domain [0,1], which is equal between any two nodes. The approximation of the second order derivative is defined as:

$$\frac{\partial^{2}\theta \left(x,F_{o}\right)}{\partial x^{2}}{|}_{x=x_{i}}=\frac{\theta_{i+1}\left(F_{o}\right)-2\theta_{i}\left(F_{o}\right)+\theta_{i-1}\left(F_{o}\right)}{h^{2}},\;\;\;1\leq i\leq l.$$

Using the central finite difference formula, Equations (14)–(17) are reduced to the following form:

(18) $$\begin{array}{c} Fo_{q}\frac{d^{2}\theta_{1}}{dFo^{2}}=-\Theta_{1}\frac{d\theta_{1}}{dF_{o}}+\frac{\left(-29\theta_{1}+38\theta_{2}-9\theta_{3}\right)}{21h^{2}}\;\\ +\frac{F_{oT}}{h^{2}}\frac{d}{dF_{o}}\left(-29\theta_{1}+38\theta_{2}-9\theta_{3}\right)+P_{f}^{2}\left(\theta_{b}-\theta_{1}\right)\times e^{a\theta_{1}}\\ +P_{m}\times 2^{\alpha \theta_{1}}+P_{r}\times exp\left(\eta_{1}\left({\bar{x}}_{1}-x_{p}\right)\right),\; \end{array}$$

(19) $$\begin{array}{c} Fo_{q}\frac{d^{2}\theta_{i}}{dFo^{2}}=-\Theta_{i}\frac{d\theta_{i}}{dF_{o}}+\frac{\left(\theta_{i+1}-2\theta_{i}+\theta_{i-1}\right)}{h^{2}}\;\\ +\frac{F_{oT}}{21h^{2}}\frac{d}{dF_{o}}\left(\theta_{i+1}-2\theta_{i}+\theta_{i-1}\right)+P_{f}^{2}\left(\theta_{b}-\theta_{i}\right)\times e^{a\theta_{i}}\\ +P_{m}\times 2^{\alpha \theta_{i}}+P_{r}\times exp\left(\eta_{1}\left({\bar{x}}_{i}-x_{p}\right)\right),\;\;1<i<n,\; \end{array}$$

(20) $$\begin{array}{c} Fo_{q}\frac{d^{2}\theta_{n}}{dFo^{2}}=-\Theta_{n}\frac{d\theta_{n}}{dF_{o}}+\frac{\left(\theta_{w}-2\theta_{n}+\theta_{n-1}\right)}{h^{2}}\;\\ +\frac{F_{oT}}{h^{2}}\frac{d}{dF_{o}}\left(\theta_{w}-2\theta_{n}+\theta_{n-1}\right)+P_{f}^{2}\left(\theta_{b}-\theta_{n}\right)\times e^{a\theta_{n}}\\ +P_{m}\times 2^{\alpha \theta_{n}}+P_{r}\times exp\left(\eta_{1}\left({\bar{x}}_{n}-x_{p}\right)\right).\; \end{array}$$

with initial conditions:

(21) $$\theta_{i}\left(0\right)=0\;\;\mathrm{and}\;\;\frac{d\theta_{i}\left(0\right)}{dF_{o}}=0,\;\;0\leq i\leq n,$$

where $\Theta_{i}=\left[1-\left(a\left(\theta_{b}-\theta_{i}\right)-1\right)F_{oq}P_{f}^{2}e^{a\theta_{i}}-\alpha F_{oq}P_{m}log\left(2\right)\times 2^{\alpha \theta_{i}}\right],\;\;1\leq i\leq n.$

### 4.2. Runge–Kutta (4,5) Scheme

Let:

(22) $$\frac{d}{dFo}\left(Fo_{q}\frac{d\theta_{i}\left(F_{o}\right)}{dFo}\right)=\frac{d\Psi_{i}\left(F_{o}\right)}{dFo}.$$

Thus, Equations (18)–(21) are reduced to the following form:

(23) $$\frac{d\theta_{i}\left(F_{o}\right)}{dFo}=\frac{\Psi_{i}\left(F_{o}\right)}{Fo_{q}},\;1\leq i\leq n,$$

(24) $$\begin{array}{c} \frac{d\Psi_{1}\left(F_{o}\right)}{dF_{o}}=-\theta_{1}\frac{\Psi_{1}\left(F_{o}\right)}{F_{oq}}+\frac{\left(-29\theta_{1}+38\theta_{2}-9\theta_{3}\right)}{21h^{2}}\;\\ +\frac{F_{oT}}{21h^{2}F_{oq}}\left(-29\Psi_{1}+38\Psi_{2}-9\Psi_{3}\right)+P_{f}^{2}\left(\theta_{b}-\theta_{1}\right)\times e^{a\theta_{1}}\\ +P_{m}\times 2^{\alpha \theta_{1}}+P_{r}\times exp\left(\eta_{1}\left({\bar{x}}_{1}-x_{p}\right)\right),\; \end{array}$$

(25) $$\begin{array}{c} \frac{d\Psi_{i}\left(F_{o}\right)}{dFo}=-\Theta_{i}\frac{\Psi_{i}\left(F_{o}\right)}{F_{oq}}+\frac{\left(\theta_{i+1}-2\theta_{i}+\theta_{i-1}\right)}{h^{2}}\;\\ +\frac{F_{oT}}{h^{2}F_{oq}}\left(\Psi_{i+1}-2\Psi_{i}+\Psi_{i-1}\right)+P_{f}^{2}\left(\theta_{b}-\theta_{i}\right)\times e^{a\theta_{i}}\\ +P_{m}\times 2^{\alpha \theta_{i}}+P_{r}\times exp\left(\eta_{1}\left({\bar{x}}_{i}-x_{p}\right)\right),\;\\ 1<i<n,\; \end{array}$$

(26) $$\begin{array}{c} \frac{d\Psi_{n}\left(F_{o}\right)}{dFo}=-\theta_{n}\frac{\Psi_{n}\left(F_{o}\right)}{Fo_{q}}+\frac{\left(\theta_{w}-2\theta_{n}+\theta_{n-1}\right)}{h^{2}}\\ +\frac{F_{oT}}{h^{2}F_{oq}}\left(-2\Psi_{n}+\Psi_{n-1}\right)+P_{f}^{2}\left(\theta_{b}-\theta_{n}\right)\times e^{a\theta_{n}}\\ +P_{m}\times 2^{\alpha \theta_{n}}+P_{r}\times exp\left(\eta_{1}\left({\bar{x}}_{n}-x_{p}\right)\right),\; \end{array}$$

with initial conditions:

(27) $$\begin{array}{c} \theta_{i}\left(0\right)=0,\mathrm{and} \end{array}$$

(28) $$\begin{array}{c} \Psi_{i}\left(0\right)=0,\; \end{array}$$

where $\Theta_{i}=\left[1-\left(a\left(\theta_{b}-\theta_{i}\right)-1\right)F_{oq}P_{f}^{2}e^{a\theta_{i}}-\alpha F_{oq}P_{m}log\left(2\right).2^{\alpha \theta_{i}}\right],$i=1,2,3,⋯,n.

The typical type of ODEs are computed with the help of the RK (4,5) technique. The proposed technique is very efficient for calculating the non-dimensional temperature of our problem. MATLAB-2018 and DEV-C++ software were used for all computational work.

## 5. Exact Solution

In order to justify the validity of the results, the exact solution is required. In the present non-linear DPLBHT model in Equation (13), when we assumed the associated blood perfusion constant a=0, associated metabolism parameter α=0, and external heat source $P_{r}=0$, then this model was reduced to the following form:

(29) $$\begin{array}{c} F_{oq}\frac{\partial^{2}\theta }{\partial F_{o}^{2}}+\left[1+F_{oq}P_{f}^{2}\right]\frac{\partial \theta }{\partial F_{o}}=\frac{\partial^{2}\theta }{\partial x^{2}}+\;F_{oT}\frac{\partial^{3}\theta }{\partial F_{o}\partial x^{2}}+P_{f}^{2}\left(\theta_{b}-\theta \right)+P_{m}, \end{array}$$

subject to the initial conditions:

(30) $$\theta \left(x,0\right)=0\;\;\mathrm{and}\;\;\frac{\partial \theta (x,0)}{\partial F_{o}}=0.$$

boundary conditions:

(31) $$\theta \left(1,Fo\right)=\theta_{w},$$

and symmetric conditions:

(32) $$\frac{\partial \theta (0,F_{o})}{\partial x}=0.$$

Taking the Laplace transform and then its inversion technique, the solution of Equation (29) under the initial condition (30) and boundary conditions (31) and (32) turns out to be:

(33) $$\begin{array}{l} \theta \left(x,F_{o}\right)=\theta_{w}\left(\frac{\cosh\left(P_{f}x\right)}{\cosh\left(P_{f}\right)}+\sum_{n=1}^{\infty }\frac{e^{S_{n1}Fo}\cosh\left(M_{Sn1}x\right)}{S_{n1}\left(S_{n1}-S_{n2}\right)}+\sum_{n=1}^{\infty }\frac{e^{S_{n2}F_{o}}\cosh\left(M_{Sn2}x\right)}{S_{n2}\left(S_{n2}-S_{n1}\right)}\right)\;\\ \;\;+\left({P_{f}}^{2}\theta_{b}+P_{m}\right)\left({P_{f}}^{-2}-\frac{\cosh\left(P_{f}x\right)}{{P_{f}}^{2}\cosh\left(P_{f}\right)}-\sum_{n=1}^{\infty }\frac{e^{S_{n1}F_{o}}\cosh\left(M_{Sn1}x\right)}{S_{n1}\left(F_{oq}{S_{n1}}^{2}+\left(1+{P_{f}}^{2}F_{oq}\right)S_{n1}+{P_{f}}^{2}\right)\left(S_{n1}-S_{n2}\right)}\right)\\ \;\;-\left({P_{f}}^{2}\theta_{b}+P_{m}\right)\left(\sum_{n=1}^{\infty }\frac{e^{S_{n2}F_{o}}\cosh\left(M_{Sn2}x\right)}{S_{n2}\left(F_{oq}{S_{n2}}^{2}+\left(1+{P_{f}}^{2}F_{oq}\right)S_{n2}+{P_{f}}^{2}\right)\left(S_{n2}-S_{n1}\right)}\right),n=1,2,3,\cdots ,\; \end{array}$$

where $r_{n}=1+{P_{f}}^{2}F_{oq}+{\left(2\;n-1/2\right)}^{2}\pi^{2}F_{oT},$$S_{n1}=\frac{-r_{n}+\sqrt{{r_{n}}^{2}-4\;F_{oq}\left({P_{f}}^{2}+{\left(2\;n-1/2\right)}^{2}\pi^{2}\right)}}{2F_{oq}},$$S_{n2}=\frac{-r_{n}-\sqrt{{r_{n}}^{2}-4\;F_{oq}\left({P_{f}}^{2}+{\left(2\;n-1/2\right)}^{2}\pi^{2}\right)}}{2F_{\mathrm{oq}}},$$M_{Sn1}=\frac{F_{oq}{S_{n1}}^{2}+\left(1+{P_{f}}^{2}F_{oq}\right)S_{n1}+{P_{f}}^{2}}{1+F_{oT}S_{n1}}$ and $M_{Sn2}=\frac{F_{oq}{S_{n2}}^{2}+\left(1+{P_{f}}^{2}F_{oq}\right)S_{n2}+{P_{f}}^{2}}{1+F_{oT}S_{n2}}.$

## 6. Results and Discussion

**Figure 2:**
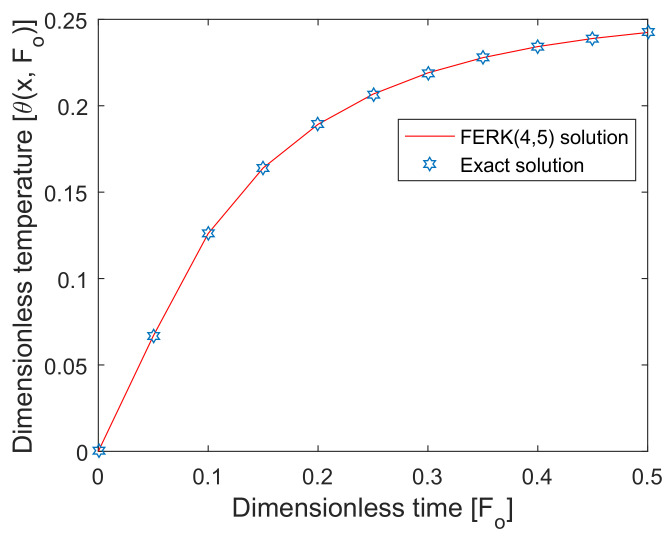
Comparison of the exact solution and the FERK (4,5) solution at x=0.9.

**Figure 3:**
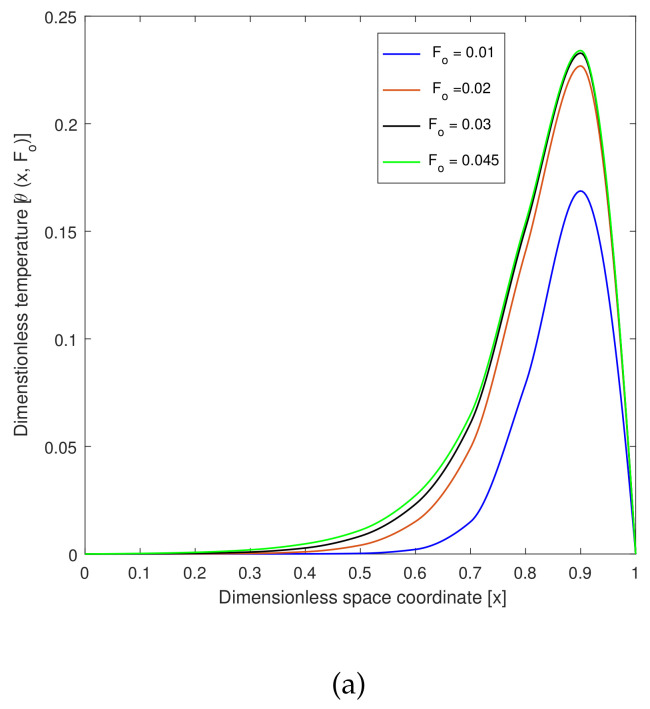

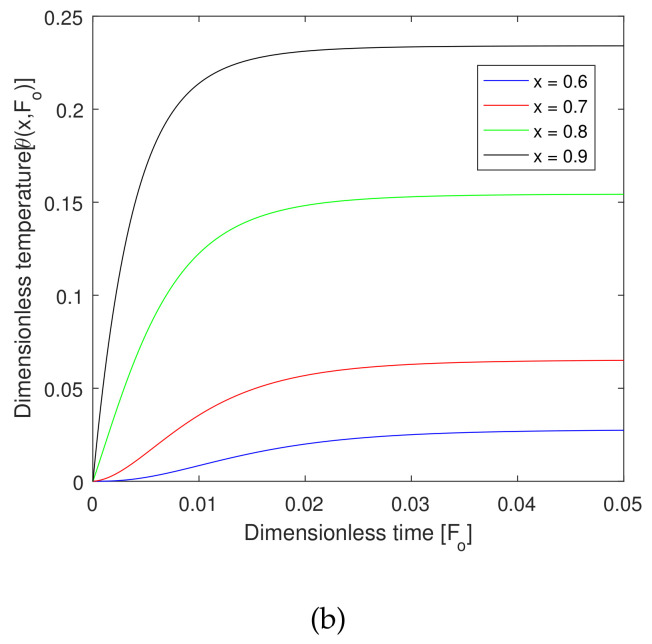
(**a**) Plot of the dimensionless temperature profile vs. the dimensionless space coordinates for different values of dimensionless time at $x_{p}=0.1$. (**b**) Plot of the dimensionless temperature profile vs. dimensionless time for different values of the dimensionless space coordinates at $x_{p}=0.1$.

**Figure 4:**
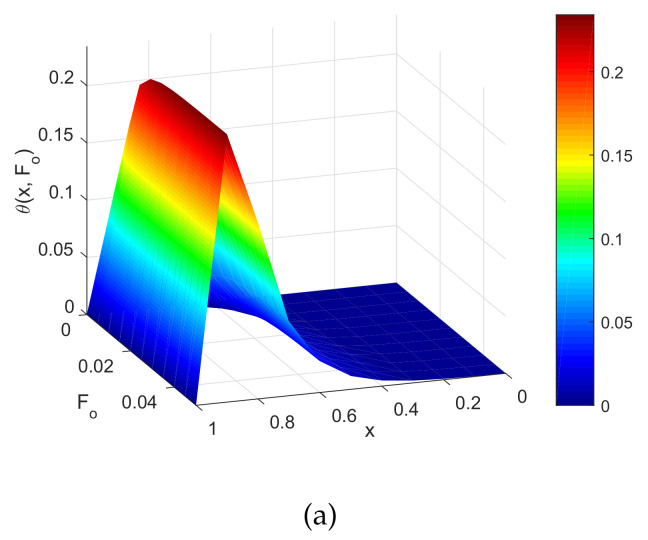

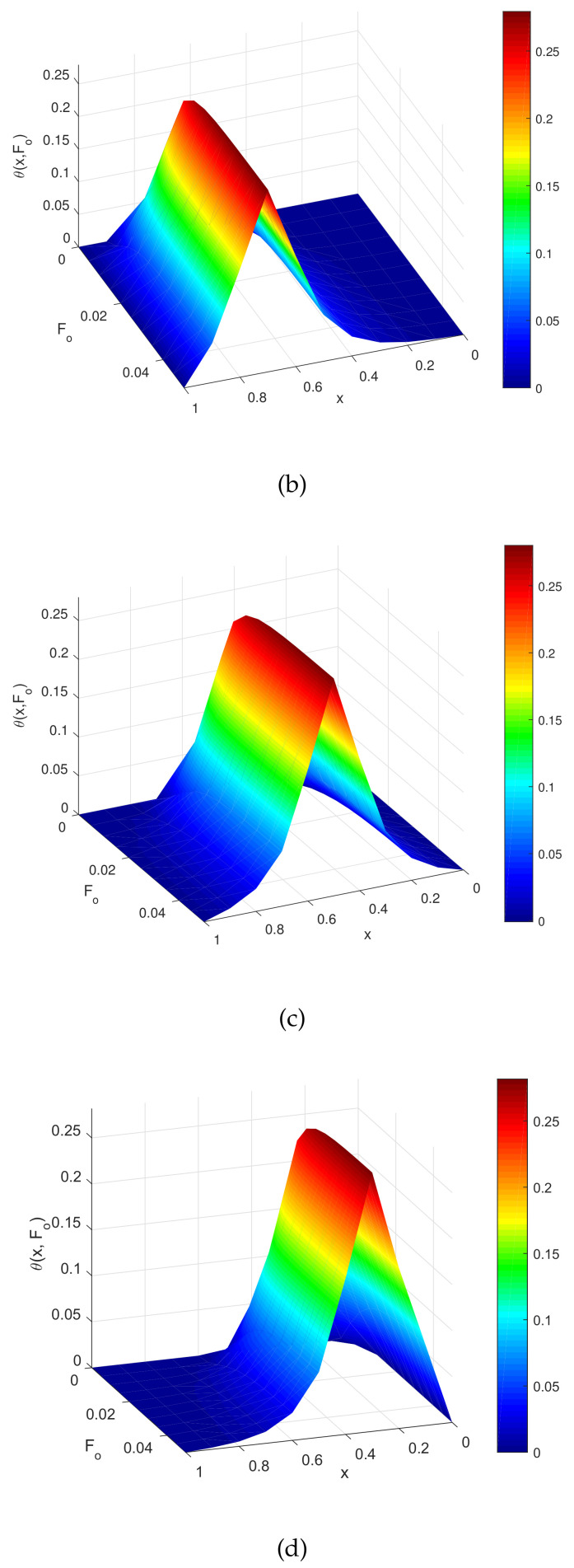

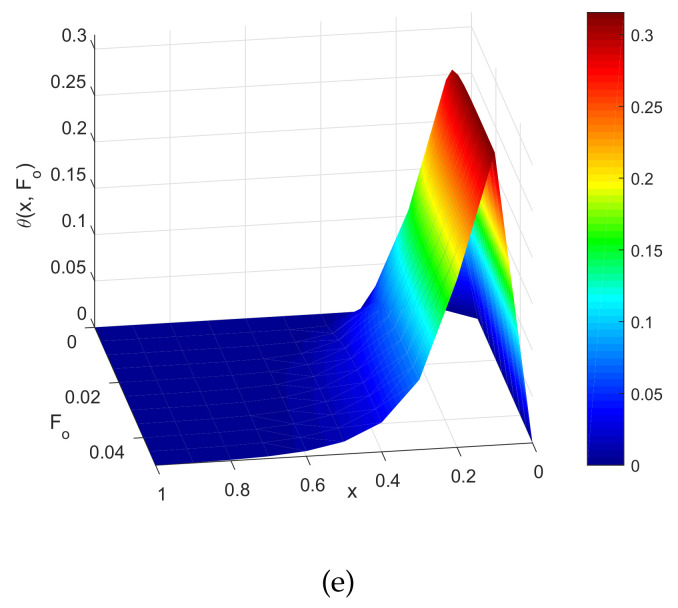
Three-dimensional plot of dimensionless temperature vs. the dimensionless space coordinates and dimensionless time for different values of the location parameters: (**a**) $x_{p}=0.1,$ (**b**) $x_{p}=0.3,$ (**c**) $x_{p}=0.5,$ (**d**) $x_{p}=0.7,$ and (**e**) $x_{p}=0.9.$

**Figure 5:**
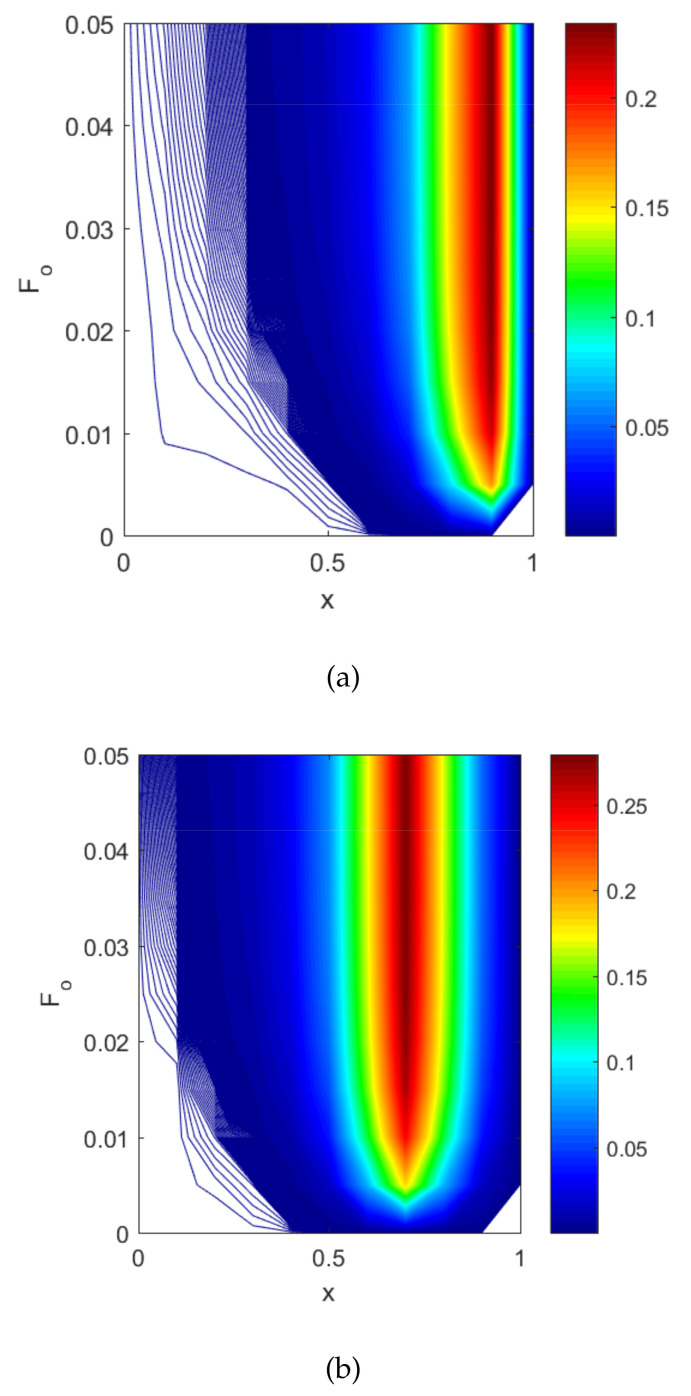

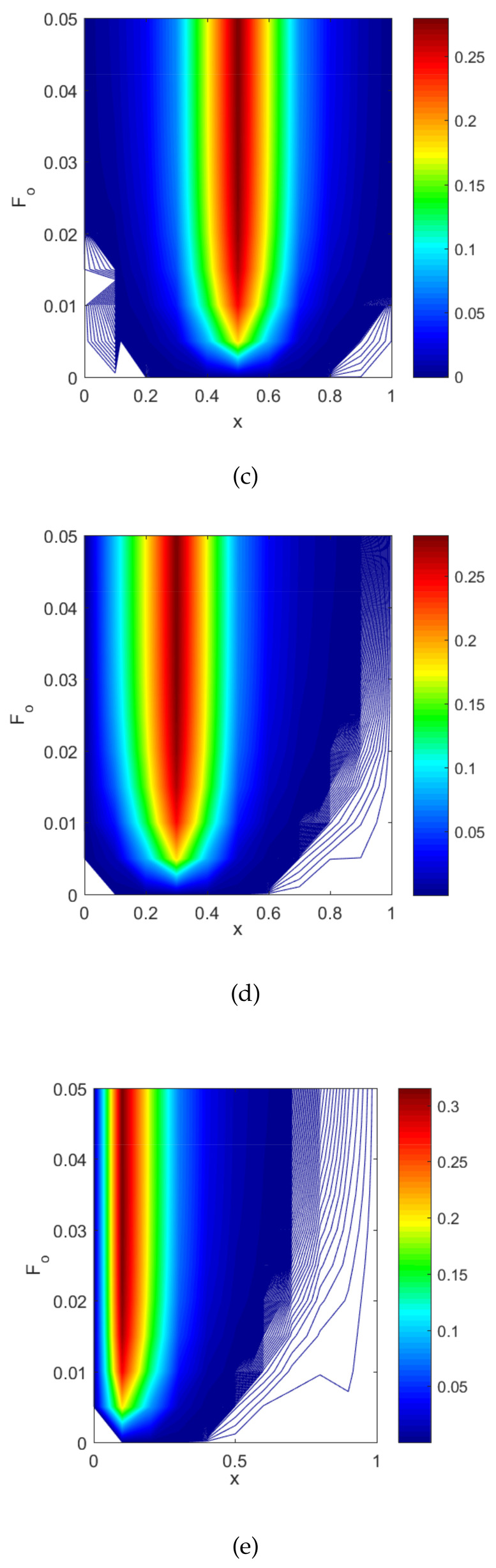
Contour plot of dimensionless temperature vs. the dimensionless space coordinates and dimensionless time for different values of the location parameters: (**a**) $x_{p}=0.1,$ (**b**) $x_{p}=0.3,$ (**c**) $x_{p}=0.5,$ (**d**) $x_{p}=0.7,$ and (**e**) $x_{p}=0.9.$

**Figure 6:**
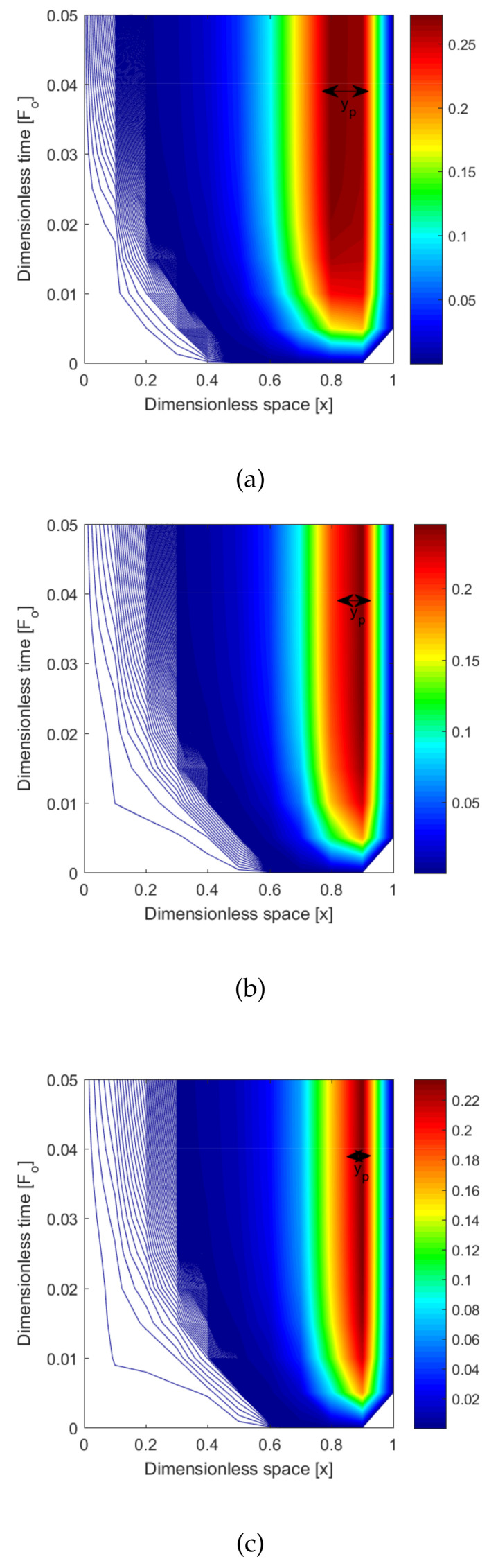
Contour plot of dimensionless temperature vs. the dimensionless space coordinates and dimensionless time for different values of cancerous region parameters: (**a**) *η* = −27, (**b**) *η* = −127, and (**c**) *η* = −227.

**Figure 7:**
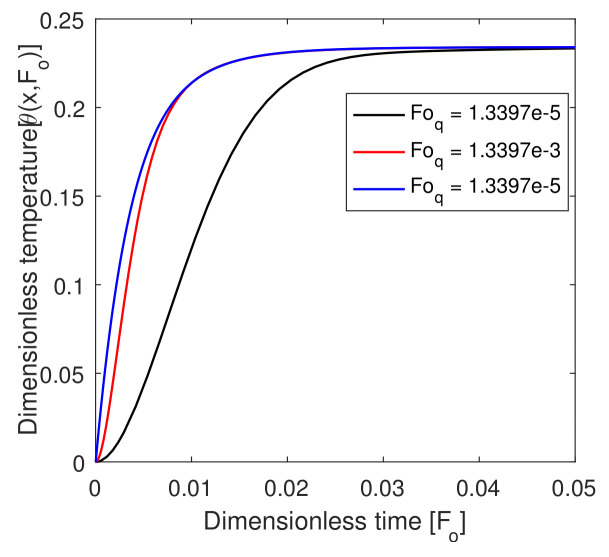
Plot of dimensionless temperature vs. dimensionless time for different values of dimensionless lag time due to heat flux parameters.

**Figure 8:**
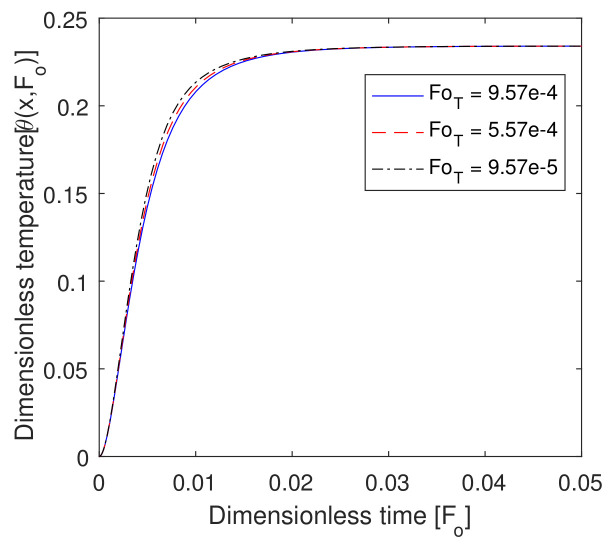
Plot of dimensionless temperature vs. dimensionless time for different values of dimensionless lag time due to the temperature gradient.

**Figure 9:**
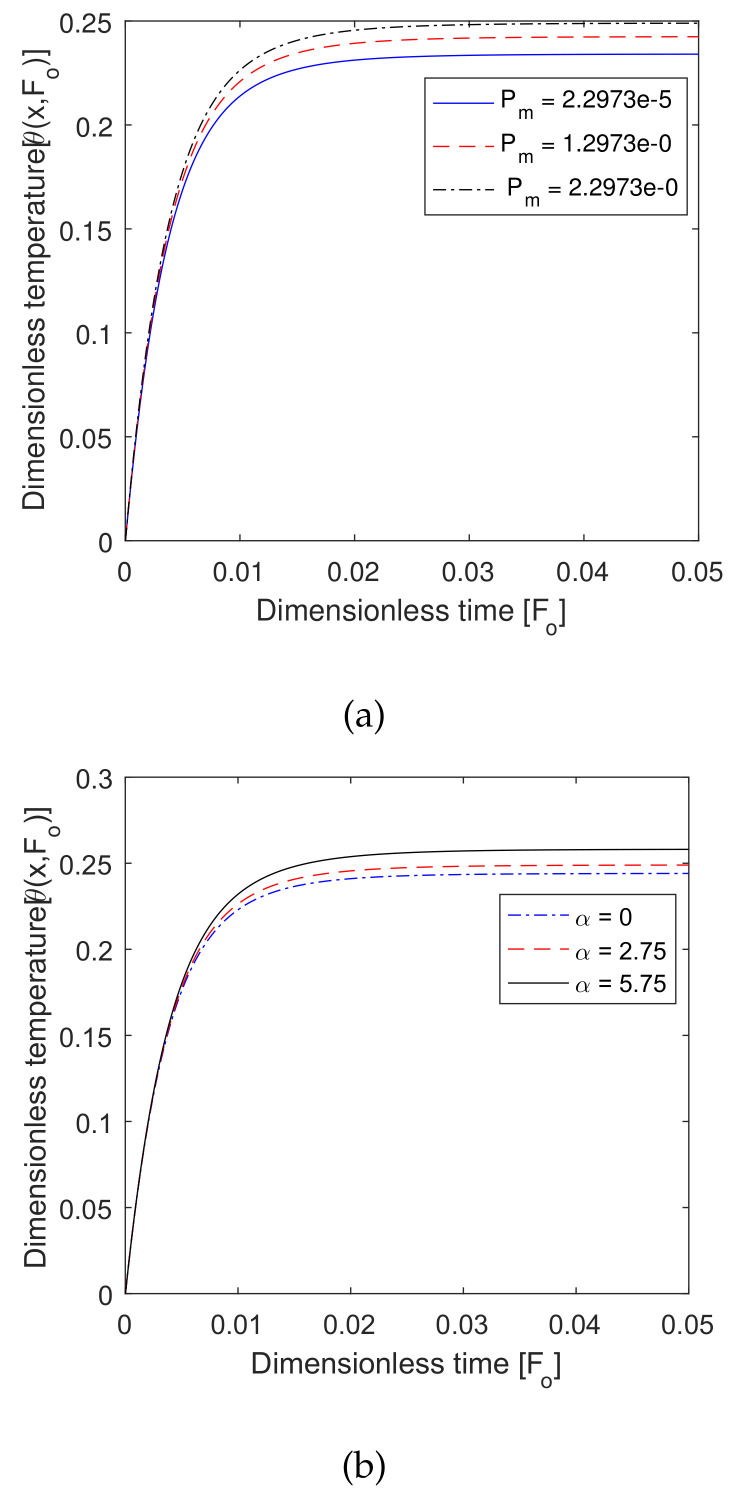
Plot of dimensionless temperature vs. dimensionless time for different values of (**a**) dimensionless metabolic heat coefficient *P_m_* and (**b**) associated metabolic heat parameter *a*.

**Figure 10:**
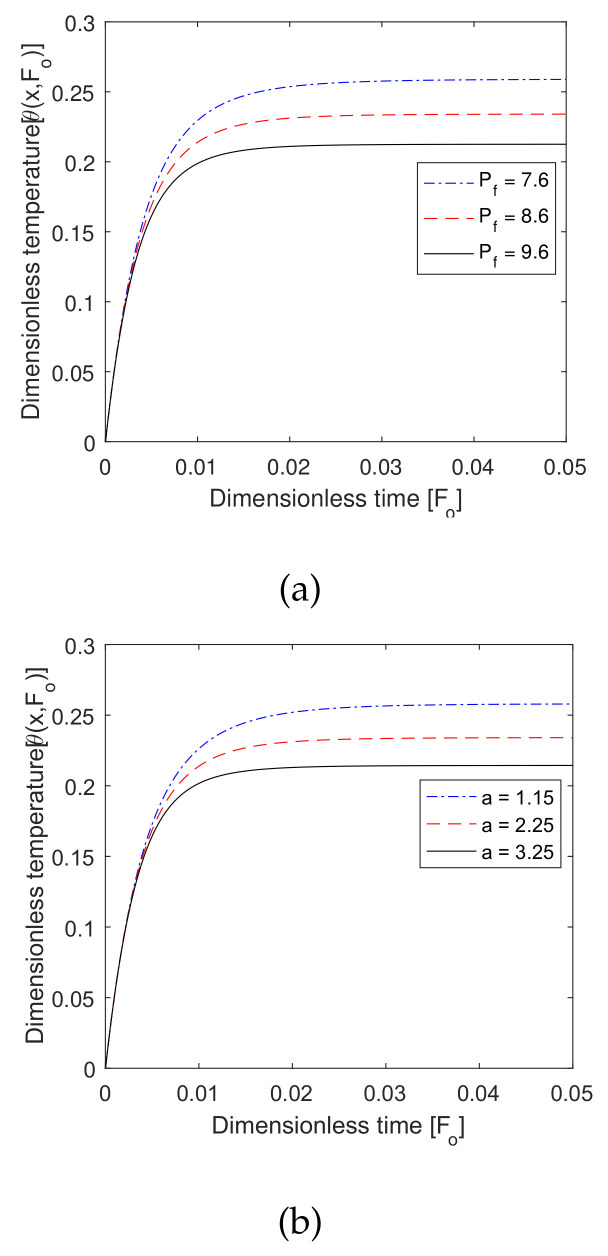
Plot of dimensionless temperature vs. dimensionless time for different values of (**a**) dimensionless blood perfusion heat source coefficient *P_f_* and (**b**) associated blood perfusion heat source parameter *a*.

**Figure 11:**
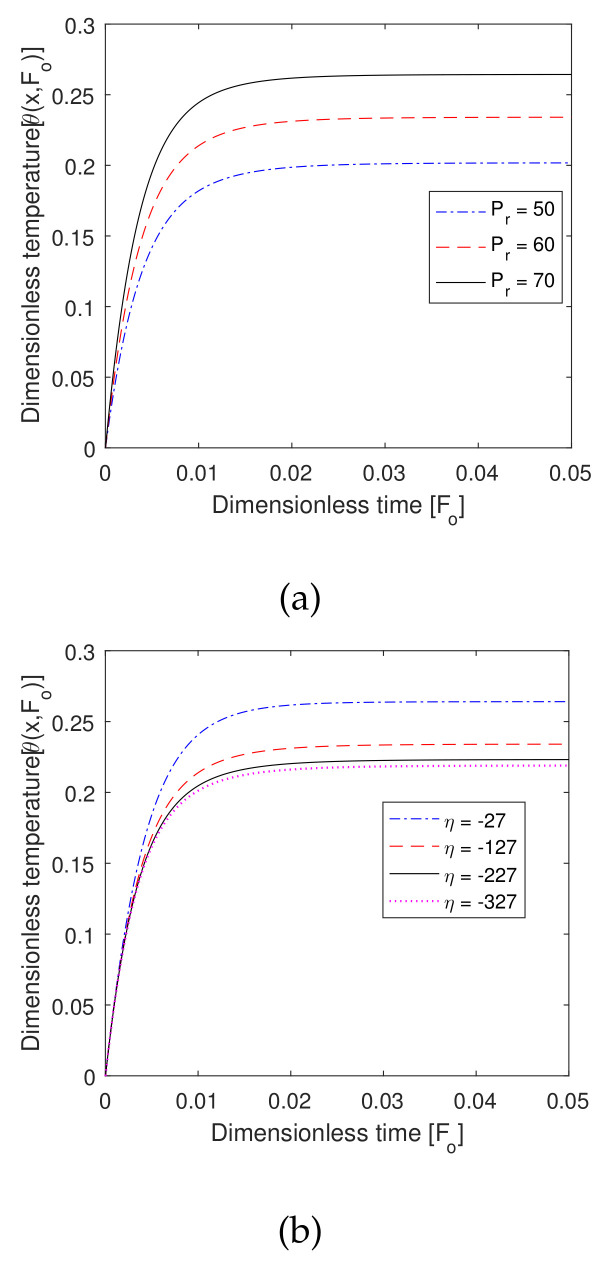
Plot of dimensionless temperature vs. dimensionless time for different values of (**a**) dimensionless external heat source coefficient *P_r_* and (**b**) associated external heat source parameter *η*.

In the proposed computational work, the temperature profile in the skin tissue was computed from the highly non-linear DPLBHT model whenever the outer boundary was kept at a fixed initial temperature. The proposed mathematical model consisted of a temperature-dependent perfusion term and also a temperature-dependent metabolic heat source, the physical function of both terms being experimentally validated. The results are shown graphically in Figure 2, Figure 3, Figure 4, Figure 5, Figure 6, Figure 7, Figure 8, Figure 9, Figure 10 and Figure 11. Those parameters whose values differed from the reference values of the non-dimensional parameters to calculate the non-dimensional temperature profile in the skin tissue with a finite length are given in the following Table 1, Table 2, Table 3, Table 4 and Table 5.

### 6.1. Comparison of the Exact and Numerical Solutions

The FERK (4,5) method was applied to find the numerical results of the proposed highly non-linear DPLBHT model. The proposed technique unified the essence of the RK (4,5) method with more efficiency and less local error [38,39]. The accurate feasibility of the present numerical scheme was shown by comparing it with the exact solution under a particular case of the non-linear DPL bioheat transfer equation in skin tissues. The comparison of the FERK (4,5) method with the exact solution is shown in Figure 2, and we observed high accuracy with less computational complexity, being merits of the proposed method.

### 6.2. Effect of $F_{o}$ and *x*

Figure 3 shows the temperature profile with respect to the dimensionless space coordinates and also with dimensionless time. Figure 3a is considered a targeted point at x=0.5. The temperature profile was approximately 0.24 ≈ 45.88 ${}^{\circ }$C, where the hyperthermia temperature lied between 41 °C and 46 °C for 15 to 60 min. Therefore, the infected or tumorous cells died for different values of $F_{o}$. The graphs are drawn for x=0.9 when dimensionless time $F_{o}=0.01$. Then, the temperature profile was less and $F_{o}=0.015$, and then, the temperature profile reached the hyperthermia temperature. Figure 3b demonstrates the temperature profile along $F_{o}$ for different values of the non-dimensional distance coordinates. The region x=0.8and0.9 reached the hyperthermia region. In this case, x=0.9 was the targeted region, so that the temperature at that position was 0.24≈45.88. Therefore, the temperature in the skin tissue achieved the hyperthermia temperature (41 ${}^{\circ }$C to 46 ${}^{\circ }$C) in the tumor region.

### 6.3. Effect of $x_{p}$

Figure 4 demonstrates three-dimensional graphs such as a,b,c,d,ande. These graphs were drawn for different values of location parameter $x_{p}$. This shows that if $x_{p}$ were changed, then the location of the tumor also changed in the skin tissue. Figure 4a–e are drawn for different values of $x_{p}=0.1,0.3,0.5,0.7,\;\mathrm{and}\;0.9.$ From this parameter, we heated the appropriate targeted point. In a similar way, we also clearly see in Figure 5 that the position of parameter $x_{p}=0.1,0.3,0.5,0.7,0.9$ was heated with the hyperthermia temperature approximately in the nbd.of 0.25 ≈ 46 ${}^{\circ }$C. From Figure 4 and Figure 5, we see that the position of hyperthermia depended on the location parameter $x_{p}$.

### 6.4. Effect of η in the Contour Plot

Figure 6 shows the effect of the regional parameter η when the values of η decreased, then the width of infected or cancerous cells decreased. From regional parameter, we confirmed accordingly the width of tumorous cells (see Figure 6). We chose the value of η according to the width of the tumor area. The regional parameter helped in the accurate heating of the cancerous cells, and the region was controlled by it. The regional parameter was very important for the hypothermia treatment of the tumor.

### 6.5. Effect of $Fo_{q}$ and $Fo_{T}$

In Figure 7, the effect of the relaxation time in heat flux is shown. The value of the temperature in the local tissue increased as dimensionless time increased from zero to 0.03 and, after that, constant at the targeted region at 0.9. Therefore, we can say that a significant effect of $Fo_{q}$ was shown in the targeted region. In Figure 8, we show the effect of lag time $Fo_{T}$ due to the temperature gradient. The value of the temperature profile decreased with the lag time $Fo_{T}$ in between dimensionless time zero to 0.3 at x=0.9. However, in comparison to $Fo_{T}$, $Fo_{q}$ was more effective in the targeted region. Therefore, $Fo_{T}$ and $Fo_{q}$ in the hyperthermia position played a vital role because the internal cells were very sensitive, and it was very effective with a small amount of temperature increase.

### 6.6. Effect of Pm and α

The effects of the metabolic heat source and its associated parameter are shown in Figure 9. In Figure 9a, we find that the value of the temperature profile increased with the metabolic heat source, and also, we observe that $P_{m}$ increased, then the temperature profile was constant. Similarly, the value of the associated metabolic coefficient increased as the temperature profile remained constant. Therefore, both Pm and α affected the hyperthermia temperature.

### 6.7. Effect of $P_{f}$

Figure 10 shows the effect of the blood perfusion coefficient and the associated blood perfusion parameter. The value of $P_{f}$ decreased as the temperature profile increased (seen in Figure 8a), and the value of α increased as the temperature profile decreased. The effects of $P_{f}$ and β were both meaningful to maintain the hyperthermia temperature at the hyperthermia position.

### 6.8. Effect of $P_{r}$ and η

In Figure 11, we see the effect of the external heat source, as well as the cancerous cells’ region parameter. We observed the value of $P_{r}$ and η to increase as the temperature profile increased (seen in Figure 9a,b). The external heat source in the cancerous cell region, the location parameter, was present in the external heat source, which was a type of Gaussian heat source. Therefore, all these parameters had a more significant effect at the position of hyperthermia.

### 6.9. Effect of Damage Integral Function (Ω)

We can see that the temperature of the skin tissue increased rapidly along with the increase in the value of reference heat source $Q_{r0}$. Therefore, the thermal damage in normal tissue may have occurred by the heating with the external heat source. The thermal damage function Ω was established by Henriques and Moritz [41], which is given as follows:

(34) $$\Omega \left(t\right)=\int_{0}^{t}A\;\exp\left(\frac{-\delta E}{R(T+273)}\right)ds,$$

where δE is the energy of the initiation of irreversible ablation; *A* is the frequency constant; *R* is the universal gas constant; and $T\;(=T_{0}+T_{0}\times \theta )$ is the local tissue temperature, which is taken from Equation (13).

Jiang et al. [40] solved Equation (34) using the difference method, and it is given as follows:

(35) $$\Omega \left(i\right)=\sum_{i=0}^{n}A\;\exp\left(\frac{-\delta E}{R(T_{0}\left(1+\theta \left(i\right)\right)+273)}\right)t,$$

where *i* indicates the time step when the node temperature was above the threshold; and *n* indicates the final time step.

Xu et al. [42] studied a formula of the probability of the thermal damage of normal tissue surrounding the tumor region, which is given as:

(36) $$P\left(i\right)=1-e^{-\Omega (i)},$$

where *P* is the probability of the thermal damage of normal tissue surrounding the tumor region.

The result was calculated when the value of the time step was 0.001 s [40], which is presented in Table 6. The numerical result showed that the thermal damage of normal tissue in the target region increased as the value of the external heat source increased. We also observe from Equation (35) that when the value of thermal damage was in the neighborhood of zero, then the probability of the normal tissue damage was near zero, i.e., no thermal damage of the normal tissue during the hyperthermia treatment.

Due to the above description, we can say that the method is very beneficial for medical science and will benefit the user.

## 7. Conclusions

A highly non-linear DPLBHT model was analyzed under the constant boundary condition in the presence of perfusion and metabolic heat sources, which were a realistic function of temperature. The external heat source was considered as a Gaussian heat source-type function, which consisted of a region of cancerous cells as a parameter, the location parameter. The results of this study are given as follows:

- The finite element RK(4,5) method was applied for the solution of the highly non-linear DPLBHT model and showed high accuracy with less computational complexity.
- The effect of the temperature profile on the different points was analyzed for a different times for the hyperthermia time.
- The effect of regional parameter η was that when the values of η decreased, then the width of the infected or cancerous cells decreased. The value of $P_{r}$ and η increased as the temperature profile increased.
- We observed that the relaxation time $Fo_{q}$ was more effective in the targeted region in comparison with the thermalization time $Fo_{T}$.
- As the value of the temperature profile increased, the metabolic heat source increased, but the perfusion rate decreased.
- Graphs were drawn for different values of location parameter $r_{p}$, then the situation of the targeted region was identified with the help of this parameter.
- We also calculated the probability of the thermal damage of the normal tissue surrounding the tumor and found no thermal damage of the normal tissue during hyperthermia treatment.

From the above observations, we concluded that the presented highly non-linear DPLBHT model played a vital role in the hyperthermia treatment of infected cells.

## Figures and Tables

**Table 1:** Physiological properties of skin tissues.

Parameters	Units	Numerical Values	References
ρ	kg/m${}^{3}$	1000	[6]
*c*	J/kg °C	4000	[32]
$c_{b}$	J/kg ${}^{\circ }$C	3340	[6]
*k*	W/m °C	0.5	[34]
*L*	*m*	0.05	[10]
$T_{0}$	°C	37	[31]
$T_{w}$	°C	37	[31]
$\tau_{q}$	s	28	[10]
$\tau_{T}$	s	20	[10]
$T_{b}$	°C	37	[32]

**Table 2:** Metabolic properties of skin tissues.

Parameters	Units	Numerical Values	References
$Q_{m0}$	W/m${}^{3}$	0.17	[11]
β	°C ${}^{-1}$	2.15	[13]

**Table 3:** Blood perfusion properties.

Parameters	Units	Numerical Values	References
$w_{b0}$	s${}^{-1}$	$3.075\times 10^{-3}$	[13]
*a*	–	2.15	[13]

**Table 4:** External heat source properties of the targeted region.

Parameters	Units	Numerical Values	References
$Q_{r0}$	W/m${}^{3}$	$5.17\times 10^{5}$	[13]
η	m${}^{-1}$	−127	[10]
$r_{p}$	m	0.005	[10]

**Table 5:** Numerical value of the parameter used in the thermal damage function.

Parameters	Units	Numerical Values	References
*A*	s${}^{-1}$	$3.11\times 10^{98}$	[15,40]
δE	J mol${}^{-1}$	$6.75\times 10^{5}$	[15,40]
*R*	J mol${}^{-1}$ K${}^{-1}$	8.314	[15,40]

**Table 6:** Thermal damage of the normal tissue during the hyperthermia treatment.

Value of $Q_{r0}$	Thermal Damage of Normal Tissue	Approximate Value of P
$5.17\times 10^{5}$	$2.4985\times 10^{-10}$	0
$5.17\times 10^{5}$	$2.8665\times 10^{-11}$	0
$5.17\times 10^{5}$	$3.2429\times 10^{-12}$	0

## Nomenclature

*T*	temperature of tissue, °C
*T_b_*	arterial blood temperature, °C
*T_w_*	wall temperature at the outer boundary, °C
*q*	heat flux, W/m^2^
*r*	space coordinates, m
*r_p_*	length of probe region, m
*L*	length of tissue, m
*t*	time, s
*τ_q_*	phase lag due to heat flux, s
*τ_T_*	phase lag due to temperature gradient, s
*c*	specific heat of tissue, J/kg °C
*c_b_*	specific heat of blood, J/kg °C
*ρ*	density of skin tissue, kg/m^3^
*k*	thermal conductivity of tissue, W/m °C
*ω_b_*_0_	reference blood perfusion term, s^−1^
*Q_m_*_0_	reference metabolic heat generation, W/m^3^
*β*	associated metabolism constant, °C^−1^
*η*	antenna constant associated with the location of the probe region, m^−1^
*δE*	energy of initiation of irreversible ablation, J mol^−1^
*A*	frequency constant, s^−1^
**Dimensionless Variables and Similarity Criteria**
*x*	dimensionless space coordinates
$F_{o}$	Fourier number or dimensionless time
$F_{o_{q}}$	dimensionless phase lag due to heat flux
$F_{o_{T}}$	dimensionless phase lag due to temperature gradient
θ	dimensionless local tissue temperature
$\theta_{b}$	dimensionless arterial blood temperature
$\theta_{w}$	dimensionless wall temperature at boundary
$P_{f}$	dimensionless blood perfusion coefficient
$P_{r}$	dimensionless external heat source coefficient
$P_{m}$	dimensionless metabolic heat source coefficient
α	associated metabolism constant
*a*	associated blood perfusion constant
Ω	thermal damage integral function

## References

[1] Siegel R., Miller K., Jemal A. (2019). Cancer Facts & Figures.

[2] Becker S.M., Kuznetsov A.V. (2014). Heat Transfer and Fluid Flow in Biological Processes.

[3] Xu F., Seffen K.A., Lu T.J. (2008). Non-Fourier analysis of skin biothermomechanics. Int. J. Heat Mass Transf..

[4] Bhowmik A., Singh R., Repaka R., Mishra S.C. (2013). Conventional and newly developed bioheat transport models in vascularized tissues: A review. J. Thermal Biol..

[5] Wang L.Q., Zhou X., Wei X. (2008). Heat Conduction: Mathematical Models and Analytical Solutions.

[6] Gupta P.K., Singh J., Rai K.N. (2013). Solution of the heat transfer problem in tissues during hyperthermia by finite difference-decomposition method. Appl. Math. Comput..

[7] Cotta R.M., Cotta B.P., Naveira-Cotta C.P., Cotta-Pereira G. (2010). Hybrid integral transforms analysis of the bioheat equation with variable properties. Int. J. Thermal Sci..

[8] Deng Z.S., Liu J. (2000). Parametric studies on the phase shift method to measure the blood perfusion of biological bodies. Med. Eng. Phys..

[9] Kim B.M., Jacques S.L., Rastegar S., Thomsen S., Motamedi M. (1996). Nonlinear finite-element analysis of the role of dynamic changes in blood perfusion and optical properties in laser coagulation of tissue. IEEE J. Sel. Top. Quant..

[10] Kumar D., Kumar P., Rai K.N. (2017). Numerical solution of non-linear dual-phase-lag bioheat transfer equation within skin tissues. Math. Biosci..

[11] Mitchell J.W., Galvez T.L., Hangle J., Myers G.E., Siebecker K.L. (1970). Thermal response of human legs during cooling. J. Appl. Physiol..

[12] Kumar P., Kumar D., Rai K.N. (2016). Non-linear dual-phase-lag model for analyzing heat transfer phenomena in living tissues during thermal ablation. J. Thermal Biol..

[13] Kumar D., Sharma N., Singh S., Rai K.N. (2018). Verified non-linear DPL model with experimental data for analyzing heat transfer in tissue during thermal therapy. Int. J. Thermal Sci..

[14] Okahara S., Miyamoto S., Soh Z., Itoh H., Takahashi S., Tsuji T. (2019). Online Prediction of Normal Blood Viscosity During Cardiopulmonary Bypass Using Hematocrit-and Temperature-Dependent Model. IEEE Access.

[15] Kumar D., Rai K.N. (2016). A study on thermal damage during hyperthermia treatment based on DPL model for multilayer tissues using finite element Legendre wavelet Galerkin approach. J. Thermal Biol..

[16] Reis R.F., Loureiro F.S., Lobosco M. (2016). 3-D numerical simulations on GPUs of hyperthermia with nanoparticles by a nonlinear bioheat model. J. Comput. Appl. Math..

[17] Andreozzi A., Iasiello M., Netti P.A. (2019). A thermoporoelastic model for fluid transport in tumor tissues. J. R. Soc. Interface.

[18] Andreozzi A., Brunese L., Iasiello M., Tucci C., Vanoli G.P. (2019). Bioheat transfer in a spherical biological tissue: A comparison among various models. IOP Conf. Ser. J. Phys. Conf. Ser..

[19] Yuan P. (2008). Numerical analysis of temperature and thermal dose response of biological tissues to thermal non-equilibrium during hyperthermia therapy. Med. Eng. Phys..

[20] Wang K., Tavakkoli F., Wang S., Vafai K. (2015). Analysis and analytical characterization of bioheat transfer during radiofrequency ablation. J. Biomech..

[21] Pennes H.H. (1948). Analysis of tissue and arterial blood temperature in the resting forearm. J. Appl. Physiol..

[22] Cattaneo C. (1958). Sur une forme de I’Equation de la chaleur elinant le paradox d’une propagation instantance. C.R. Acad. Sci..

[23] Vernotte M.P. (1958). Les paradoxes de la theorie continue de I equation de la chleur. C. R. Acad. Sci..

[24] Tzou D.Y. (1995). A unified field approach for heat conduction from micro to macroscale. J. Heat Transf..

[25] Tzou D.Y. (1996). Macro-to-Microscale Heat Transfer: The Lagging Behavior.

[26] Kumar P., Kumar D., Rai K.N. (2015). A numerical study on dual-phase-lag model of bio-heat transfer during hyperthermia treatment. J. Thermal Biol..

[27] Mochnacki B., Majchrzak E. (2017). Numerical model of thermal interactions between cylindrical cryoprobe and biological tissue using the dual-phase lag equation. Int. J. Heat Mass Transf..

[28] Klinger H.G. (1983). Relation between heat transfer in perfused biological tissue and the local symmetry components of the vascular system. J. Math. Biol..

[29] Pal D.S., Pal S. (1993). Effects of blood flow, curved boundary and environmental conditionson temperature distribution in a two dimensional modelof human skin and subcutaneous tissue. J. Math. Biol..

[30] Zhang Z.W., Wang H., Qin Q.H. (2014). Method of fundamental solutions for nonlinear skin bioheat model. J. Mech. Med. Biol..

[31] Kengne E., Lakhssassi A., Vaillancourt R. (2012). Temperature Distributions for Regional Hypothermia Based on Nonlinear Bioheat Equation of Pennes Type: Dermis and Subcutaneous Tissues. Appl. Math..

[32] Saedodin S., Noroozi M.J., Ganji D.D. (2015). A New Solution of Nonlinear Bio-Heat Transfer Equation in Living Tissues Under Periodic Heat Flux in Tissue Surface. J. Biomater. Tissue Eng..

[33] Saedodin S., Ganji D.D., Noroozi M.J. (2015). Investigation of Nonlinear Models of Heat Transfer in Hyperthermia Therapy of Pancreas Tissue. J. Biomater. Tissue Eng..

[34] Hassanpour S., Saboonchi A. (2017). Validation of local thermal equilibrium assumption in a vascular tissue during interstitial hyperthermia treatment. J. Mech. Med. Biol..

[35] Do T.N., Haas Z.J. (2020). On the Design of RD-MIMO: Spatial Multiplexing versus Opportunistic Transmission Schemes. IEEE Access.

[36] Habash R.W., Bansal R., Krewski D., Alhafid H.T. (2006). Thermal therapy, part 2: Hyperthermia techniques. Crit. Rev. Biomed. Eng..

[37] Salloum M., Ma R.H., Weeks D., Zhu L. (2008). Controlling nanoparticle delivery in magnetic nanoparticle hyperthermia for cancer treatment: Experimental study in agarose gel. Int. J. Hyperthermia.

[38] Strikwerda J.C. (1989). Finite Difference Schemes and Partial Differential Equations.

[39] Bogacki P., Shampine L.F. (1996). An Efficient Runge–Kutta (4,5) Pair. Comput. Math. Appl..

[40] Jiang S.C., Ma N., Li H.J., Zhang X.X. (2002). Effects of thermal properties and geometrical dimensions on skin burn injuries. BURN.

[41] Henriques F.C., Moritz A.R. (1947). The conduction of heat to and through skin and the temperature attained there in a theoretical and an experimental investigation. Am. J. Pathol..

[42] Xu F., Wang P.F., Lin M., Lu T.J., Ng E.Y.K. (2010). Quantifying the underlying mechanism of skin thermal damage: A review. J. Mech. Med. Biol..

